# HCMV Targets the Metabolic Stress Response through Activation of AMPK Whose Activity Is Important for Viral Replication

**DOI:** 10.1371/journal.ppat.1002502

**Published:** 2012-01-26

**Authors:** Jessica McArdle, Nathaniel J. Moorman, Joshua Munger

**Affiliations:** 1 Department of Biochemistry and Biophysics, University of Rochester Medical Center, Rochester, New York, United States of America; 2 Department of Microbiology and Immunology, University of North Carolina, Chapel Hill, North Carolina, United States of America; University of Pennsylvania, United States of America

## Abstract

Human Cytomegalovirus (HCMV) infection induces several metabolic activities that have been found to be important for viral replication. The cellular AMP-activated protein kinase (AMPK) is a metabolic stress response kinase that regulates both energy-producing catabolic processes and energy-consuming anabolic processes. Here we explore the role AMPK plays in generating an environment conducive to HCMV replication. We find that HCMV infection induces AMPK activity, resulting in the phosphorylation and increased abundance of several targets downstream of activated AMPK. Pharmacological and RNA-based inhibition of AMPK blocked the glycolytic activation induced by HCMV-infection, but had little impact on the glycolytic pathway of uninfected cells. Furthermore, inhibition of AMPK severely attenuated HCMV replication suggesting that AMPK is an important cellular factor for HCMV replication. Inhibition of AMPK attenuated early and late gene expression as well as viral DNA synthesis, but had no detectable impact on immediate-early gene expression, suggesting that AMPK activity is important at the immediate early to early transition of viral gene expression. Lastly, we find that inhibition of the Ca^2+^-calmodulin-dependent kinase kinase (CaMKK), a kinase known to activate AMPK, blocks HCMV-mediated AMPK activation. The combined data suggest a model in which HCMV activates AMPK through CaMKK, and depends on their activation for high titer replication, likely through induction of a metabolic environment conducive to viral replication.

## Author Summary

Human Cytomegalovirus (HCMV) is a ubiquitous human pathogen that is a major cause of birth defects. HCMV can also cause severe disease in immunocompromised individuals including transplant recipients, leukemia patients and those infected with HIV. It is clear that upon infection, HCMV takes control of numerous cellular processes that are important for the virus to generate the next round of infectious virions. We have previously found that upon infection, HCMV reprograms the metabolic activity of the host-cell. Here, we find that this metabolic reprogramming largely depends on the viral activation of a cellular protein called the AMP-activated protein kinase (AMPK). AMPK is a central regulator of cellular energy production that is typically only activated when cellular energy stores are very low. Our results indicate that HCMV-mediated activation of AMPK is necessary to flip the metabolic switch thereby driving host-cell metabolic activation and viral replication. As inhibition of AMPK blocked viral replication, and had little impact on uninfected host-cell metabolism, targeting AMPK could have therapeutic potential to treat HCMV-associated disease.

## Introduction

Upon infection, viruses must create a cellular environment conducive to viral replication. While there are many different aspects of this virally-induced environment, a critical component of this cellular reprogramming is the diversion of cellular resources such as energy and molecular building blocks to the production of viral progeny. Numerous viruses, ranging from small, non-enveloped RNA viruses to large enveloped DNA viruses have been reported to target the host-cell metabolic machinery [1]–[9]. This suggests that cellular metabolic function is a key virus-host interaction.

Human cytomegalovirus (HCMV), a member of the betaherpesvirus family, is a major cause of birth defects upon congenital infection as well as morbidity in immunosuppressed populations [10]–[12]. HCMV has been found to cause drastic changes to the host cell metabolic network upon infection, increasing the concentrations of select glycolytic enzymes, the steady state levels of glycolytic metabolites, and the fluxes through glycolysis and the TCA cycle [13]–[14]. Though the exact mechanisms through which HCMV induces glycolytic activation are not clear, it has recently been shown that virally-mediated activation of glycolysis can be blocked through inhibition of CaMKK and that HCMV infection induces the expression of the Glut4 transporter [15]–[16]. Here we explore the role of the AMP-activated protein kinase (AMPK) during HCMV replication and HCMV-mediated glycolytic activation.

AMPK is a heterotrimeric, serine-threonine kinase that functions as a major energy regulator for the cell. Low ATP levels result in increased concentrations of AMP through the action of adenylate kinase [17]. These increased AMP concentrations induce AMP binding to AMPK and subsequent stimulation of AMPK activity, primarily through either LKB1 or CaMKK-dependent phosphorylation [18]–[21]. Upon activation, AMPK works to restore the ATP pool by activating ATP-producing pathways while simultaneously inhibiting ATP-consuming pathways [22]–[23]. One pathway positively regulated by AMPK is glycolysis. Upon AMPK activation, glycolysis can be upregulated through several mechanisms. AMPK targets numerous key glycolytic enzymes including glucose transporters (Glut1 and Glut4), hexokinase and PFK-2, to increase glycolytic flux [23]–[26].

Here we show that AMPK activity is increased throughout viral infection relative to mock-infected fibroblasts. Additionally, high-titer HCMV replication requires activated AMPK as pharmaceutical or RNAi-based inhibition of AMPK severely attenuates the production of viral progeny. Consistent with a role in glycolytic activation, AMPK inhibition also leads to an attenuation of HCMV-induced glycolytic flux. These results suggest that AMPK is a critical cellular protein targeted by HCMV infection.

## Results

### AMPK activity increases upon HCMV infection

**Figure 1:**
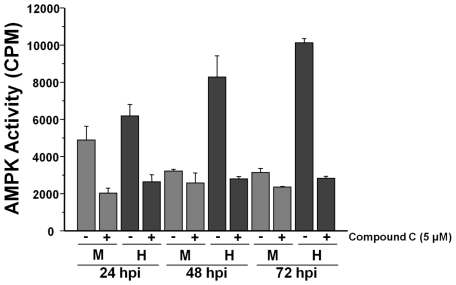
AMPK is activated during HCMV infection. Serum-starved MRC-5 human fibroblasts were mock- infected or infected with HCMV (MOI = 3). After adsorption, cells were treated with DMSO or the AMPK inhibitor, Compound C (5 µM). At 24, 48 and 72 h post infection cells were harvested in lysis buffer and the resulting lysates were assayed for AMPK activity using the SAMS peptide as a substrate. Values are mean + SE (n = 2).

To determine if AMPK might be responsible for the metabolic induction observed during HCMV infection, we analyzed mock or HCMV-infected extracts for AMPK activity using a well described *in vitro* AMPK activity assay [27]. To help verify the specificity of the measured AMPK activity, we also performed assays in the presence of Compound C, a specific AMPK inhibitor [28]. At 24 h post-infection both mock and HCMV-infected cells exhibited similar amounts of AMPK activity (Fig. 1). Compound C treatment suppressed the observed AMPK activity to a background level of ∼2000 CPM, which was a consistent background level at all time points examined (Fig. 1). At later time points, i.e. 48 and 72 h post-infection, the AMPK activity associated with mock-infected fibroblasts fell significantly to background levels (Fig. 1). In contrast, the AMPK activity associated with HCMV-infected lysates increased over this time frame with much greater AMPK activity levels observed in HCMV-infected lysates than in mock-infected lysates at the same time points (Fig. 1). These results indicate that HCMV induces AMPK activity upon infection.

**Figure 2:**
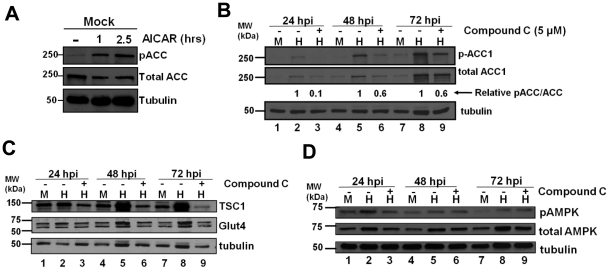
Impact of AMPK inhibition on AMPK substrate accumulation during HCMV infection. (**A**) MRC-5 human fibroblasts were incubated in glucose free media with DMSO (-) or AICAR for 1h or 2.5 h prior to blotting with antibodies specific for pSer79-specific ACC1, total ACC1, or tubulin. (**B-D**) Serum-starved MRC-5 human fibroblasts were mock infected or infected with HCMV (MOI = 3). After adsorption, cells were treated with the AMPK inhibitor, Compound C (5 µM), or DMSO (-). Cells were harvested at 24, 48 and 72 h post infection and analyzed by Western blot with antibodies specific for pSer79-specific ACC1, total ACC1 and tubulin in (**B**), TSC1, Glut4 and tubulin in (**C**), and pThr172-specific AMPK, total AMPK and tubulin (**D**). Relative pACC/ACC signal ratios in (**B**) were estimated during HCMV infection for DMSO and Compound C-treated cells using densitometry and subsequently normalized to the ratio of the DMSO control sample.

Previously we have found that HCMV infection increases the total levels of the fatty acid biosynthetic enzyme, acetyl-CoA carboxylase (ACC1) as well as the amount of phosphorylated ACC1 [29]. AMPK has been shown to phosphorylate ACC1 at Ser79, resulting in decreased ACC1 activity with the end-result being inhibition of fatty acid biosynthesis and conservation of ATP [17]. To determine if activated AMPK increases levels of phosphorylated ACC1 in MRC-5 fibroblasts, we treated cells with an AMPK activator, AICAR [30]. AICAR treatment resulted in substantial increases in the abundance of phosphorylated ACC (Fig. 2A), consistent with activation of AMPK. Subsequently, we analyzed the levels of total ACC1 and Ser79 phosphorylated ACC1 in the presence of the AMPK inhibitor, Compound C, during HCMV infection. At 24 h post-infection, HCMV-infected lysates contained more Ser79-phosphorylated ACC1 than mock-infected lysates (Fig. 2B). At this time, treatment with Compound C reduced the levels of Ser79-phosphorylated ACC1 to the levels found in uninfected cells, indicating that AMPK might be responsible for the increased abundance of phosphorylated ACC1 (Fig. 2B). At 48 and 72 h post-infection, treatment with Compound C reduced the levels of Ser79-phosphorylated ACC1 in HCMV infected lysates but also decreased the levels of total ACC1 (Fig. 2B). Using densitometry, the relative signal ratios of pACC to ACC in the HCMV infected lysates were examined (Fig. 2B). Treatment with Compound C had the largest impact on the pACC/ACC ratio at 24 hpi, reducing it ∼10-fold. At subsequent times post infection, Compound C reduced the pACC/ACC ratio at every time point, consistent with decreased phosphorylation, albeit to a much lesser extent than observed at 24 hpi (Fig. 2B). While the decreases in phospho-ACC1 relative to total ACC1 upon Compound C treatment are consistent with inhibition of AMPK-mediated phosphorylation of ACC1, Compound C treatment also impaired the HCMV-induced accumulation of total ACC1 suggesting that Compound C treatment could be impacting normal HCMV infection.

Previous reports indicate that HCMV infection induces the levels of the Glut4 glucose transporter and the tuberous sclerosis protein (TSC1), a negative regulator of mTOR signaling [15], [31]. Activated AMPK has been shown to increase Glut4 expression [32] as well as TSC1 levels through prevention of proteosome mediated TSC1 degradation [33]–[34]. Our results indicating that HCMV infection induces AMPK activity suggest the possibility that the induction of these proteins during HCMV infection may result from increased AMPK activity. As previously reported [31], in the absence of inhibitor treatment, HCMV infection has little impact on TSC1 levels at 24 h post-infection but significantly increased the levels of TSC1 at 48 h post-infection and 72 h post-infection (Fig. 2C). Treatment of HCMV-infected cells with Compound C substantially reduced the levels of TSC1 at all time points compared to DMSO treated controls (Fig. 2C) suggesting that AMPK activity is necessary for HCMV-mediated induction of TSC1 levels. As was reported previously [15], we also observed increases in Glut4 in HCMV-infected cells as compared to mock-infected cells at 48 and 72 h post-infection (Fig. 2C). Treatment with Compound C inhibited this induction of Glut4 levels (Fig. 2C) suggesting that AMPK activity is important for the viral induction of Glut4 expression. Taken together, our results indicate that HCMV-infection activates AMPK which in turn is necessary for the induction of TSC1 and Glut4 levels.

AMPK can be activated by phosphorylation at residue Thr172 mediated by either LKB1 or CaMKK [17]. As shown in Figure 2D, HCMV-infected extracts contained a higher level of total and phosphorylated AMPK then mock extracts at 24, 48 and 72 h post-infection. In both mock and HCMV-infected cells, the amount of Thr172-phosphorylated AMPK appeared to be the greatest at 24 h post-infection and subsequently declined as infection progressed (Fig. 2D). For uninfected cells, this decline in Thr172-phosphorylated AMPK correlated with the decreased AMPK activity observed in cellular lysates (Fig. 2D). For the HCMV-infected cells, the increase in AMPK activity observed as infection progressed did not correlate with the levels of Thr172-phosphorylated AMPK although the levels of total AMPK remained elevated. This combination of increased abundance of total AMPK and increased AMPK Thr172 phosphorylation likely contribute to the observed increases in AMPK activity during HCMV infection, although as AMPK is reported to be regulated by multiple phosphorylation events [35]–[37], other mechanisms of activation cannot be ruled out. Treatment of cells with Compound C did not appreciably impact AMPK Thr172 phosphorylation (Fig. 2D), not surprising given that Compound C inhibits AMPK through competitive inhibition at its ATP binding site [28]. Taken together, our results suggest that HCMV activates AMPK during infection likely in part due to an increase in both the phosphorylation at Thr172 as well as the total abundance of AMPK.

### Inhibition of AMPK attenuates HCMV-mediated glycolytic activation

**Figure 3:**
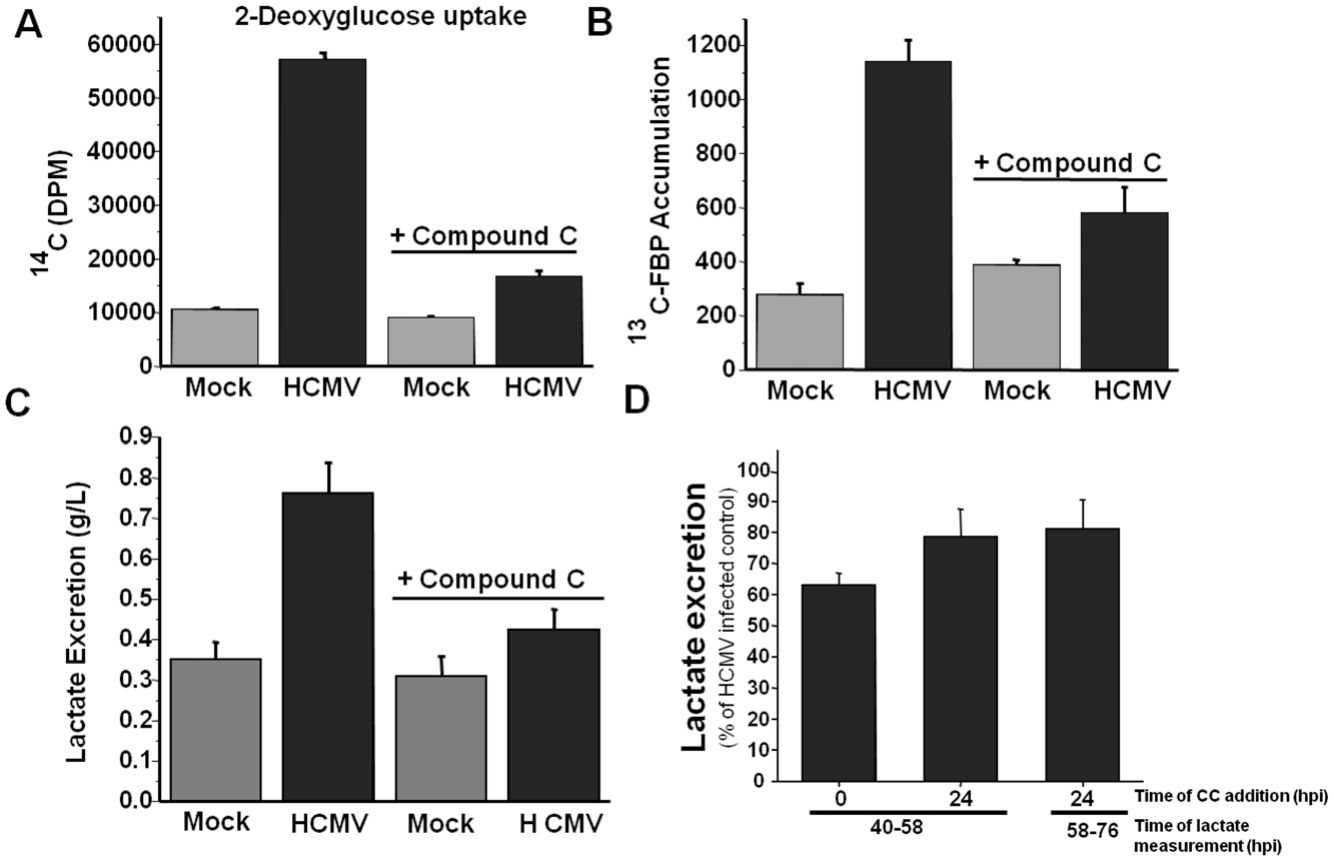
Inhibition of AMPK blocks HCMV-induced glycolytic activation. Serum-starved MRC-5 human fibroblasts were mock infected or infected with HCMV (MOI = 3). After adsorption, cells were treated with the AMPK inhibitor Compound C (5 µM) or DMSO. (**A**) At 48 h post infection, cells were labeled with [^14^C]-deoxyglucose for 5 min and harvested for scintillation counting as described in the materials and methods. (**B**) At 48 h post infection, cells were labeled for 1 min with [^13^C] glucose containing DMEM, quenched with cold methanol, and processed for LC-MS/MS to measure the accumulation of [^13^C] fructose bisphosphate. All values are means + SE (n = 2). (**C**) At 40 h post infection, fresh media was added to all cells. At 58 h post infection, media was removed from mock and HCMV-infected fibroblasts and lactate secretion into the media over the 18 hr time frame was analyzed. All values are means + SE (n = 6). (**D**) Serum-starved MRC-5 human fibroblasts were HCMV infected (MOI = 3) and Compound C (5 µM) was added either immediately after adsorption (0) or 24 h post infection. Lactate excretion was measured from 40–58 hpi or from 58–76 hpi. Values are shown as the percent of the HCMV-infected, DMSO treated control and are means + SE (n≥3).

AMPK is a central metabolic regulator whose activation can activate glycolysis by targeting multiple steps within the glycolytic pathway including glucose uptake and phosphofructokinase activity [17]. To determine if AMPK is important for the induction of glucose import by HCMV, we treated mock or HCMV-infected fibroblasts with Compound C and analyzed glucose import using a radioactive glucose analog. Consistent with previous reports [15], [26], HCMV infection induced glucose uptake greater than 5-fold compared to mock-infected cells (Fig. 3A). Treatment with Compound C almost completely reversed this increase (Fig. 3A). Compound C had a negligible impact on glucose uptake in mock-infected fibroblasts (Fig. 3A). These data indicate that HCMV relies heavily on AMPK activity to activate glucose import whereas AMPK is not critical for glucose uptake in uninfected fibroblasts.

To further analyze how activation of AMPK contributes to HCMV-mediated glycolytic activation, we measured the rate of glycolytic labeling after treatment with Compound C using ^13^C-labeled glucose as a metabolic tracer. Specifically, utilizing LC-MS/MS we measured the rate of ^13^ C-fructose bisphosphate accumulation, a central glycolytic metabolite, after pulse with ^13^C-glucose. Treatment with Compound C led to an approximate 2-fold decrease in ^13^C-labeled FBP accumulation in HCMV-infected fibroblasts (Fig. 3B). In contrast, inhibition of AMPK had little impact on the labeling rate of mock-infected fibroblasts (Fig. 3B). Lastly, we measured how inhibition of AMPK impacted the most downstream glycolytic phenotype, accumulation of lactate in the media. Treatment with Compound C substantially reduced lactate secretion in HCMV-infected fibroblasts, but not mock-infected fibroblasts (Fig. 3C). In all of the glycolysis assays tested, the inhibition of glycolytic flux upon Compound C treatment was specific for HCMV-infected cells. This suggests that AMPK is important for HCMV-induced glycolytic activation, but does not contribute appreciably to the glycolytic rate in uninfected fibroblasts, which is consistent with AMPK's described role as a stress-induced metabolic regulator [17].

Addition of Compound C immediately following adsorption blocked HCMV-mediated activation of glycolysis (Fig. 3A–C). To determine whether this glycolytic activation was sensitive to AMPK inhibition after the establishment of infection, we treated cells with Compound C at 24 hpi, a time at which immediate early gene expression is peaking, early genes are being expressed and viral DNA replication is initiating [38]. Subsequently, we measured lactate excretion into the media from 40-58 hpi, and from 58–76 hpi. As compared to a DMSO-treated control, treatment with Compound C immediately following adsorption resulted in a ∼40% reduction in lactate excretion (Fig. 3D). Treatment of cells at 24 hpi resulted in a ∼20% reduction in lactate excretion whether measured from 40–58 or 58–76 hpi (Fig. 3D). These results suggest that while HCMV-mediated activation of glycolysis is more sensitive to AMPK inhibition at the very beginning of infection, the induction of glycolysis is still attenuated when AMPK is inhibited during an HCMV infection that has already been established.

### Inhibition of AMPK attenuates production of HCMV viral progeny

**Figure 4:**
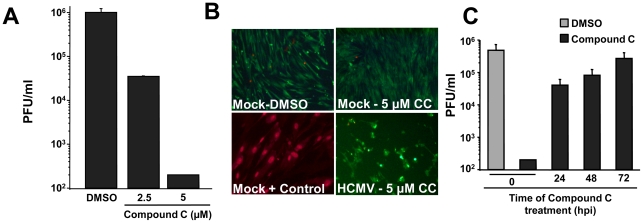
Pharmaceutical inhibition of AMPK attenuates HCMV viral replication. (**A**) Serum-starved MRC-5 human fibroblasts were mock-infected or infected with HCMV (MOI = 3). After adsorption, cells were treated with the AMPK inhibitor, Compound C (CC), at concentrations of 2.5 µM or 5 µM or with DMSO alone. Cells were harvested at 96 h post infection and the production of infectious viral progeny was measured by a standard plaque assay. Values are means + SE (n = 3). (**B**) Analysis of the potential toxicity of Compound C treatment. Confluent MRC-5 fibroblasts were mock infected or infected with HCMV (MOI = 3). After adsorption, cells were treated with 5 µM Compound C (CC). At 72 h post infection, cell viability was measured via a Live/Dead cell viability assay. Green indicates the presence of esterase activity associated with viable cells, while red indicates loss of cellular membrane integrity associated with cell death. As a positive control for staining of a breakdown in membrane integrity, cells were briefly treated with ethanol. (**C**) Serum-starved MRC-5 human fibroblasts were HCMV-infected (MOI = 3). Compound C was added at adsorption (0), 24, 48 or 72 h post infection. As a viral growth control, DMSO was added to HCMV infected cells at adsorption. All samples were harvested at 96 h post infection and the production of infectious viral progeny was measured by a standard plaque assay. Values are means + SE (n = 2).

Given that HCMV-infection induces AMPK activity (Fig. 1), and that AMPK activity is important for HCMV-mediated glycolytic activation (Fig. 3), we next tested if AMPK inhibition impacts viral replication. We treated fibroblasts with DMSO or two different concentrations of Compound C, and analyzed the production of viral progeny by plaque assay. Treatment with increasing concentrations of Compound C resulted in a dose-dependent decrease in viral titers. A greater than 20-fold defect in production of viral progeny was observed at 2.5 µM Compound C and a greater than 1000-fold defect was observed in cells treated with 5 µM Compound C (Fig. 4A). To exclude the possibility of toxicity from drug treatment, we also performed a Live/Dead assay which stains live cells green based on their esterase activity and stains the nucleic acids of dead cells red based on the breakdown of membrane integrity. Treatment with Compound C at the highest concentration (5 µM, Fig. 4B) resulted in little to no red staining in both mock- and HCMV-infected fibroblasts ( <1%) with ubiquitous green staining (>99%) suggesting that Compound C treatment is not toxic to MRC-5 fibroblasts up to a concentration of 5 µM. These results suggest that AMPK activity is important for HCMV replication and that inhibition of AMPK does not induce significant toxicity in MRC-5 fibroblasts.

We were interested in determining at what point during the infectious cycle AMPK activity might be required for high-titer HCMV replication. To address this issue, we treated cells with Compound C at various points post-infection and analyzed viral replication. Addition of Compound C at 24 and 48 hrs resulted in a greater than 10 and 5-fold reduction in the production of viral progeny, respectively, compared to DMSO treated (Fig. 4C). These are significant reductions in viral yield and suggest that AMPK is important throughout the duration of HCMV infection for peak viral production. However, the difference in viral growth between treatment at adsorption and treatment at 24 h is large (∼100-fold, Fig. 4C), suggesting that the major requirement for AMPK activity occurs during the first 24 h of infection. Addition of Compound C at 72 hrs post-infection had a negligible impact on HCMV viral growth (Fig. 4C). This suggests that AMPK activity is not necessary during the late stages of growth. Furthermore, this lack of inhibition when added at 72 hpi suggests that Compound C is not blocking infectious HCMV production through some artifactual interaction with newly produced HCMV virions.

### Impact of pharmaceutical AMPK inhibition on the viral life cycle

**Figure 5:**
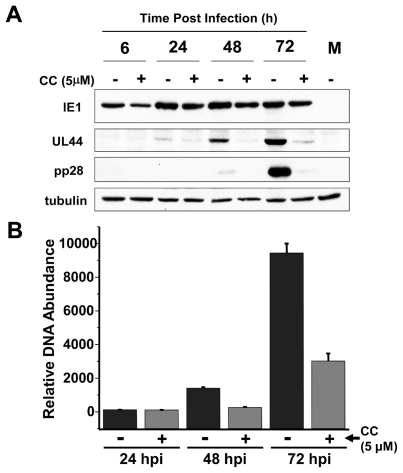
Impact of AMPK pharmaceutical inhibition on viral protein and DNA accumulation. (**A**) Analysis of the impact of AMPK inhibition on viral protein accumulation. Serum-starved MRC-5 human fibroblasts were mock infected or infected with HCMV (MOI = 3). After adsorption, cells were treated with the AMPK inhibitor Compound C (CC, 5 µM) or DMSO alone (-). Cells were harvested at 6, 24, 48 and 72 h post infection and analyzed by Western blotting with antibodies specific for IE1, UL44, pp28 and tubulin. (**B**) Analysis of the impact of AMPK inhibition on viral DNA accumulation. Serum-starved MRC-5 cells were mock infected or infected with HCMV (MOI = 3). After adsorption, cells were treated with the AMPK-specific inhibitor Compound C (CC, 5 µM) or DMSO. Viral DNA was extracted from cells that were harvested at 24, 48, and 72 h post infection and processed for qPCR analysis of viral DNA accumulation. Values are means + SE.

With the observation that Compound C treatment decreases HCMV viral titers, we were interested in investigating how this inhibition impacted other aspects of the viral life cycle. We performed Western blot analysis and quantitative real-time PCR (qPCR) to determine the effects of Compound C on viral protein accumulation and viral DNA replication, respectively. The expression of the immediate early protein, IE1, was largely unaffected by Compound C treatment (Fig. 5A), however a large decrease in the abundance of the early protein U_L_44 and the late protein pp28 was observed (Fig. 5A). These results suggest that AMPK is required for the transition from the immediate early to the early stages of infection. As many early genes are involved in the regulation of viral DNA replication, we hypothesized that this defect in early gene expression could result in decreased viral DNA replication. As shown in Figure 5B, when virally infected fibroblasts are treated with Compound C, a marked decrease in viral DNA accumulation is observed, most notably at 48 and 72 h post-infection. These findings suggest that AMPK inhibition affects the viral life cycle at early stages of infection and inhibits viral DNA replication.

### RNAi-mediated AMPK inhibition attenuates HCMV replication and HCMV-mediated activation of glycolysis

**Figure 6:**
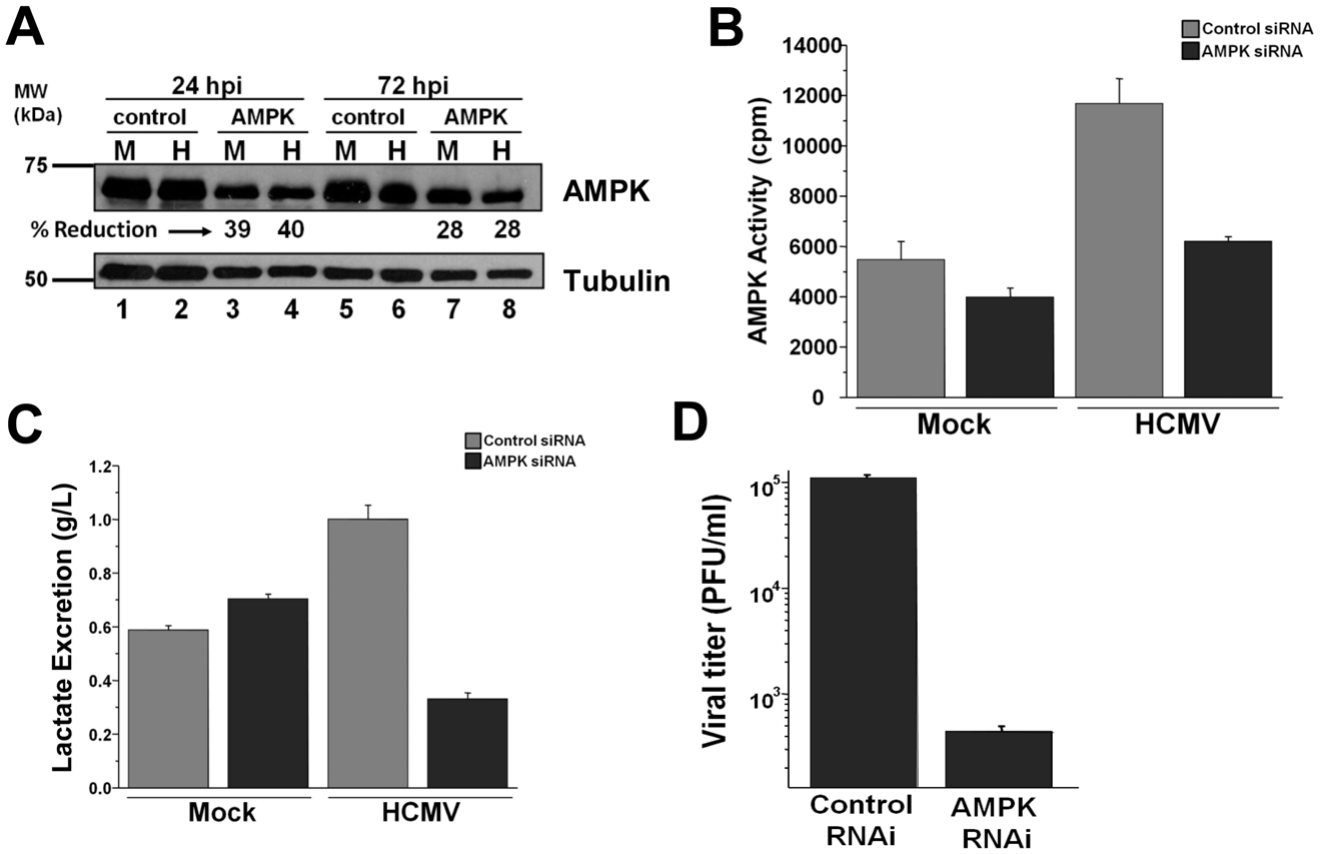
RNAi-mediated targeting of AMPK inhibits HCMV replication. MRC-5 fibroblasts were transfected with control or AMPK-specific siRNA. Fibroblasts were then serum-starved at 48 h post transfection and subsequently mock or HCMV-infected 24 h later. (**A**) Samples were harvested for Western blot analysis of AMPK knockdown. The RNAi-mediated percent reduction of AMPK expression from the relevant mock or HCMV-infected control sample was estimated using densitometry. (**B**) At 48 h post infection, samples were harvested in lysis buffer and cellular lysates were assayed for AMPK activity by measuring phosphorylation of the SAMS peptide by ^32^P-ATP. Results are shown as mean + SE (n = 3). (**C**) At 40 h post infection, fresh media was added to all cells. At 58 h post infection, media was removed from mock and HCMV-infected fibroblasts and lactate secretion into the media over the 18 hr time frame was analyzed. All values are means + SE (n = 6). (**D**) At 96 h post infection, HCMV-infected samples transfected with either control or AMPK-specific siRNA were harvested for viral plaque assay to measure viral replication. Results are shown as mean + SE (n = 2).

The finding that pharmaceutical inhibition of AMPK attenuates viral replication and HCMV-induced glycolytic flux suggests that AMPK plays an important role during viral infection. Despite reports that Compound C is a specific inhibitor of AMPK [28], the possibility for off-target effects is always an issue with pharmaceutical inhibitors. To confirm the importance of AMPK for HCMV replication, we employed an AMPK-specific RNAi to decrease AMPK expression during infection. Transfection of RNAi specific for AMPK resulted in an ∼40% reduction in AMPK abundance in both mock and HCMV-infected fibroblasts at 24 h post-infection in comparison to control RNAi transfected cells (Fig. 6A). Analysis of AMPK activity indicated that transfection of AMPK-specific RNAi prior to HCMV infection reduced AMPK activity by approximately 50%, comparable to mock levels (Fig. 6B). AMPK-specific RNAi had a much smaller impact on the AMPK activity of mock-infected cells (Fig. 6B), consistent with a relative lack of AMPK activity in mock-infected cells to start with. Analysis of media lactate accumulation indicated that AMPK-specific RNAi ablated the HCMV-mediated induction of lactate excretion, but had little impact on the lactate excretion of mock-infected cells (Fig. 6C). Analysis of how RNAi-mediated AMPK inhibition impacted viral replication indicated a greater than 250-fold reduction in viral progeny production as compared to control cells (Fig. 6D). In total, these results confirm our findings that AMPK is a critical cellular factor required both for HCMV-mediated glycolytic induction as well as for high-titer replication.

### The impact of CaMKK inhibition on AMPK activity

**Figure 7:**
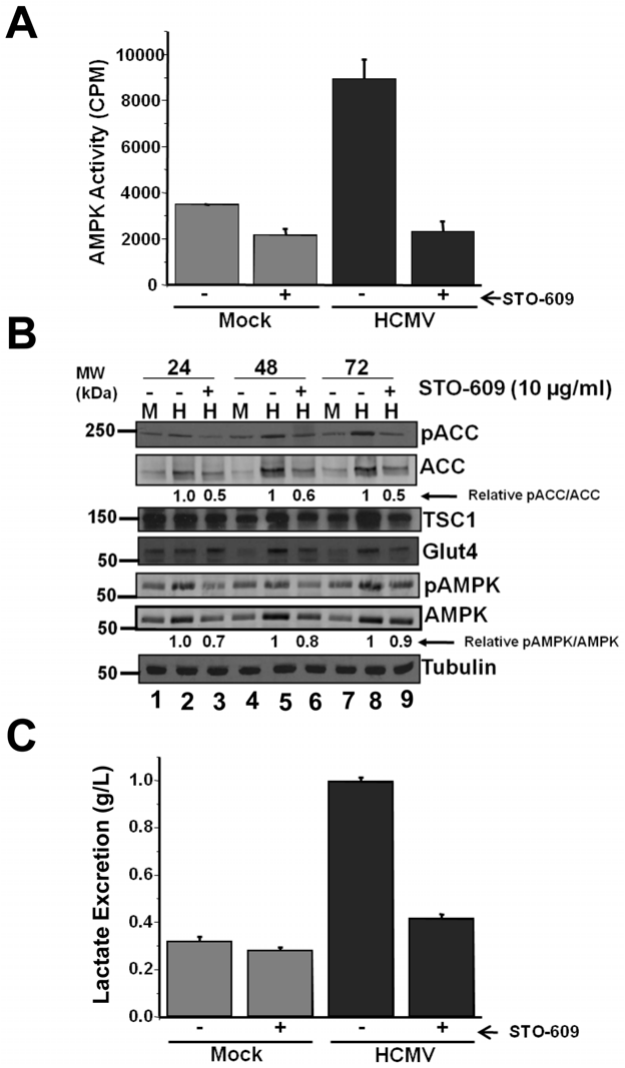
Impact of CaMKK inhibition on AMPK substrates. Serum-starved MRC-5 human fibroblasts were mock infected or infected with HCMV (MOI = 3). After adsorption, cells were treated with the CaMKK inhibitor STO-609 (10 µg/ml) or DMSO (-). At 24, 48 and 72 h post infection cells harvested in lysis buffer and the resulting lysates were assayed for AMPK activity using the SAMS peptide as a substrate (**A**) or analyzed by Western blotting with antibodies specific for Ser79-pACC, ACC, TSC1, Glut4,Thr172-pAMPK, AMPK and tubulin (**B**). Relative pACC/ACC and Thr172-pAMPK/AMPK signal ratios in (**B**) were estimated during HCMV infection for DMSO and Compound C-treated cells using densitometry and subsequently normalized to the DMSO control. (**C**) At 40 h post infection, fresh media was added to all cells. At 58 h post infection, media was removed from mock and HCMV-infected fibroblasts and lactate secretion into the media over the 18 hr time frame was analyzed. All values are means + SE (n = 6).

Previously, we have shown that glycolysis is upregulated upon HCMV infection, and that CaMKK appears to be required for both productive viral replication and virally-induced glycolytic flux [16]. CaMKK has been shown to be involved in the regulation of AMPK activity under various conditions [18], [39]. Given the observation that AMPK appears to be important for both productive viral replication and HCMV-induced glycolytic flux as well, it seemed likely that CaMKK could be responsible for activating AMPK during HCMV infection. In order to determine the importance of CaMKK for AMPK activation, we used the CaMKK-specific inhibitor STO-609 to treat fibroblasts and subsequently analyzed the impact on AMPK activity. As shown in Figure 7A, inhibition of CaMKK blocked the induction of AMPK activity during HCMV infection. Importantly, it has been previously reported that STO-609 treatment at a similar dosage does not impact AMPK activity directly or affect AMPK activation induced by another AMPK-activating kinase, LKB1 [19], [40].

To further explore the impact of CaMKK inhibition on AMPK activity, we examined the accumulation and phosphorylation of AMPK and its substrates by Western blot after treatment with STO-609. Pharmaceutical inhibition of CaMKK decreased the levels of phosphorylated AMPK, but also reduced the levels of total AMPK (Fig. 7B). Analysis of the relative ratio of phospho-AMPK to total AMPK indicated STO-609 treatment shifted the ratio towards the unphosphorylated AMPK by 30% at 24 hpi (Fig. 7B). The observed reductions in total as well as pAMPK upon STO-609 treatment likely contribute to the reduction in AMPK activity observed in STO-609-treated cells (Fig. 7A). Analysis of Ser79 phosphorylated-ACC upon STO-609 treatment indicated a similar trend. The amounts of Ser79-phosphorylated ACC and total ACC were both reduced upon STO-609 treatment, with a reduction of 40-50% in the relative pACC/ACC ratio. Similar to the observation with AMPK inhibition, treatment with STO-609 also blocked the increases in TSC1 and Glut4 levels observed during HCMV-infection (Fig. 7B). As we had previously analyzed the impact of STO-609 treatment on HCMV activated ^13^C-FBP labeling [16], we wanted to extend these observations with respect to measuring lactate production. Consistent with our previous ^13^C-FBP labeling results, STO-609 treatment blocked the induction of lactate secretion associated with HCMV infection yet had no effect on the accumulation of lactate production in mock-infected cells (Fig. 7C). Taken together, our results demonstrate that inhibition of CaMKK inhibits HCMV-mediated AMPK activation as well as the accumulation and phosphorylation of downstream AMPK targets which is consistent with a model in which CaMKK mediates AMPK activation during HCMV infection.

## Discussion

**Figure 8:**
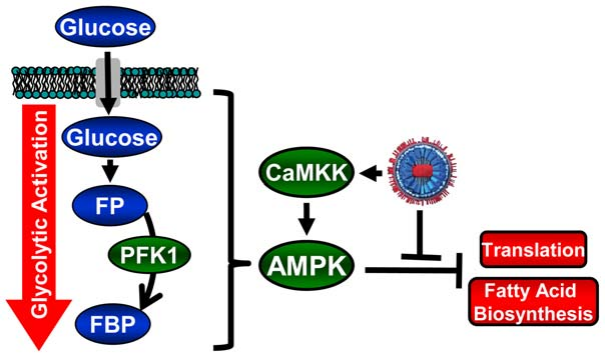
Model of HCMV-mediated manipulation of the AMPK pathway. HCMV infection results in AMPK activation, likely through activation of CaMKK. AMPK activation results in glycolytic activation to provide energy and building blocks necessary for production of viral progeny. AMPK activation would typically result in inhibition of cellular processes important for viral replication, e.g. protein translation and fatty acid biosynthesis. Viral gene products block these consequences of AMPK activation, e.g. U_L_38 binds TSC complex and prevents inhibition of mTOR and protein translation. (FP =  fructose-6-phosphate, FBP  =  fructose-1,6-bisphosphate).

We have previously established that HCMV infection induces numerous changes to the host-cell metabolic network [13]–[14]. Induction of glycolysis has also been found to be critical for high-titer HCMV replication [16], [41]. Here we report that HCMV activates AMPK, a metabolic stress kinase, and that HCMV depends on its activity for high-titer replication. HCMV requires AMPK activation to increase glucose import and drive increased glycolytic flux (Fig. 8). Inhibition of AMPK attenuated both early and late gene expression and markedly reduced viral DNA replication. These results suggest that AMPK is an important cellular factor for HCMV replication.

Unstressed, uninfected cells do not normally utilize AMPK to activate glycolysis [17]. Our results support this view, as the inhibition of AMPK did not impact the import of glucose or the FBP labeling rate in uninfected cells (Fig. 3). In contrast to uninfected cells, HCMV infection induces the activation of AMPK, which is critical for HCMV-mediated glycolytic activation. Interestingly, activation of AMPK would be predicted to have several consequences that are detrimental to infection including inhibition of protein translation and fatty acid biosynthesis [42]. AMPK-mediated inhibition of translation occurs through induction of the TSC1/2 complex which in turn negatively regulates translation through inhibition of mTOR [33]–[34]. It has recently been shown that the HCMV U_L_38 protein can bind to the TSC1/2 complex and prevent its inhibitory activity on mTOR and translation initiation [31], [43]. Taken together, it appears that HCMV infection induces AMPK activation which in turn drives glycolytic activation, yet blocks the anti-viral effects of AMPK activation through the action of specific gene products such as U_L_38 (Fig. 8).

While the U_L_38 protein appears sufficient to block the inhibitory effects of AMPK activation on mTOR activity, it is less clear how HCMV infection blocks AMPK's inhibitory effects on fatty acid biosynthesis. We have previously found that HCMV induces fatty acid biosynthesis, and specifically induces the activity of acetyl-CoA carboxylase (ACC), the rate-limiting enzyme of fatty acid biosynthesis [14], [29]. ACC, and consequently fatty acid biosynthesis, is negatively regulated by activated AMPK [17]. Given that HCMV requires activated fatty acid biosynthesis and ACC activity for viral replication, it is likely that HCMV infection blocks the negative impact of activated AMPK on ACC activity, potentially through the activity of an HCMV viral protein.

Our results suggest that inhibition of CaMKK blocks the down-stream effects associated with activated AMPK (Fig. 7). Previous reports suggest that pharmaceutical inhibition of CaMKK using STO-609 blocks CaMKK-mediated activation of AMPK but does not impact LKB1- mediated AMPK activation or activation of AMPK upon energetic stress, for example, upon treatment with glycolysis inhibitors [19], [40]. Taken together, these results suggest that HCMV infection requires CaMKK activity to activate AMPK, though the exact mechanism responsible is unclear. Our results suggest that inhibition of CaMKK reduces the amount of Thr172-phosphorylated AMPK, a known CaMKK phosphorylation site, as well as the total amount of AMPK. Both of these effects would be predicted to contribute to a decrease in AMPK activity during HCMV infection. Other AMPK phosphorylation sites have been implicated in the regulation of AMPK activity [35]–[37], thus CaMKK could potentially be modulating AMPK activity through phosphorylation of sites other than Thr172 as well.

We have previously reported that inhibition of CaMKK blocks high-titer virus production [16]. Our current findings that AMPK inhibition blocks HCMV replication to a similar extent as CaMKK inhibition is consistent with a model in which HCMV-mediates AMPK activation through CaMKK (Fig. 8). How HCMV infection induces CaMKK activity still remains to be determined, although it has previously been reported that HCMV infection induces Ca^2+^ release from ER stores and we have found that HCMV infection induces CaMKK expression [16], [44], both of which would be predicted to increase CaMKK activity.

Glycolysis has been shown to be important for HCMV replication, and glycolytic inhibition has a similar impact on HCMV as inhibition of AMPK and CaMKK [16], [41]. The similarity is both quantitative, in terms of the magnitude of reduction in viral titers, as well as qualitative, in blocking viral DNA replication and late gene expression, attenuating early gene expression and having no detectable impact on immediate early gene expression [16], [41]. Given these similarities, and combined with the observed necessity of AMPK and CaMKK for HCMV-induced glycolysis, the simplest model would be that CaMKK and AMPK activation are important for HCMV replication due to their activation of glycolysis. Despite these correlations, it remains to be determined how much of HCMV's reliance on CaMKK and AMPK activity is due to their activation of glycolysis. The possibility that these kinases contribute to viral infection through phosphorylation of other cellular or viral targets cannot be ruled out.

In summary, we find that HCMV infection activates AMPK, which is required for HCMV-mediated glycolytic activation and high-titer HCMV replication. As AMPK activation signals metabolic stress to normal cells, HCMV has evolved mechanisms to block the anti-viral consequences of metabolic stress pathway activation. While some of these mechanisms are known, such as U_L_38 maintaining mTOR activation through interaction and inhibition of the TSC complex, others remain to be elucidated, such as maintenance of fatty acid biosynthesis. This stress response balancing act is representative of a recurrent theme in virus-host evolution. Replicating viruses must create a cellular environment conducive to viral replication, the efforts of which host cells have evolved to resist. In the case of AMPK, the evolutionary struggle is for the keys to the host-cell metabolic machinery. As the AMPK pathway is not normally activated in uninfected cells and inhibition of AMPK activity is tolerated in animal models [45]–[46], targeting this pathway clinically could be therapeutically beneficial for preventing HCMV-associated disease.

## Materials and Methods

### Cell culture and virus infection

MRC-5 fibroblasts were cultured in Dulbecco modified Eagle medium (DMEM; Invitrogen) supplemented with 7.5% fetal bovine serum. Cells were grown to confluence in either 10 cm or 6-well tissue culture plates. Once confluent, medium was removed and serum-free DMEM was added. Cells were maintained in serum-free medium for 24 h prior to infection.

HCMV (strain Ad169) was used to infect cells at a multiplicity of infection (MOI) of 3 for all of the current experiments. Mock-infected controls were treated with an equal volume of medium containing the same serum concentrations as virus-treated cells. Virus adsorptions were carried out for 90 min at 37°C, after which viral innocula were aspirated and serum-free DMEM was added back. Production of infectious virus was measured by standard viral plaque assay.

### Chemical reagents

STO-609 (EMD Biosciences), a specific inhibitor of CaMKK and Compound C (Calbiochem), a specific inhibitor of AMPK, were maintained in DMSO at concentrations of 5 mg/ml at −20°C and 10 mg/ml at 4°C, respectively.

### Kinetic flux profiling

Labeled DMEM was prepared from glucose-free media by adding 10 mM HEPES and either labeled (^13^C) or unlabelled (^12^C) glucose to a final concentration of 4.5 gL^-1^. For flux analysis, samples were switched to fresh, unlabelled medium 24 h and 1 h before final addition of ^13^C-labeled medium. Samples were labeled for 1 min and the reaction was quenched by the addition of 4 ml −80°C 80% methanol and incubation at −80°C for 10 minutes. Cells were then scraped in the methanol, centrifuged at 3000 rpm for 5 min at 4°C and the supernatant was collected. The pellet was extracted twice more in 500 µl cold methanol, adding the resulting supernatants to the previously collected supernatant. After extraction, the supernatants were dried down under nitrogen gas and resuspended in 175 µl of 50% methanol. Samples were subsequently spun down at full speed for 5 min at 4°C and the remaining supernatant was transferred to HPLC sample vials.

### Liquid chromatography-tandem mass spectrometry analysis

The accumulation of fully-^13^C-labeled fructose 1,6-bisphosphate was monitored using liquid chromatography-tandem mass spectrometry (LC-MS/MS) as previously described [14] and is briefly discussed below. LC-MS/MS was performed using a LC-20AD HPLC system (Shimadzu) and a Synergi Hydro-RP column (150×2 mm with a 5 µm-particle size; Phenomenex) coupled to a mass spectrometer. The LC parameters were as follows: autosampler temperature, 4°C; injection volume, 20 µl; column temperature, 40°C; flow rate, 15 µl/sec. The LC solvents were solvent A, 100% methanol; solvent B, 10 mM tributylamine and 15 mM acetic acid in 97:3 water:methanol. The gradient conditions were as follows: negative mode—t = 0, 100% B; t = 5, 100% B; t = 10, 80% B; t = 20, 80% B; t = 35, 35% B; t = 38, 5% B; t = 42, 5% B; t = 43, 100% B; t = 50, 100% B. Mass spectrometric analyses were performed on a TSQ Quantum Ultra triple-quadrupole mass spectrometer running in multiple reaction monitoring mode (MRM) (Thermo Fisher Scientific). Peak heights for fructose-1,6-bisphosphate-extracted ion chromatograms were analyzed using Excalibur software (Thermo Fisher Scientific).

### Western Blot analysis

Proteins from cell lysates were solubilized in 1X disruption buffer (50 mM Tris (pH 7.0), 2% SDS, 5% 2-mercaptoethanol, and 2.75% sucrose), separated by 10% SDS-PAGE and transferred to nitrocellulose in Tris-glycine transfer buffer. Blots were stained with Ponceau S to visualize protein and ensure equal sample loading. The membranes were blocked in 5% milk in TBST followed by incubation in primary antibody. After subsequent washes, blots were incubated in secondary antibody and protein bands were visualized using the ECL detection system (Pierce). Antibodies used were specific for the following viral proteins: IE1 (Shenk Laboratory, unpublished), U_L_44 (Virusys), and pp28 [47] and the following cellular proteins: tubulin (Epitomics), TSC1 (Millipore), Glut4 (Abcam), ACC and phosphor-Ser79-ACC (Cell Signaling Technologies), AMPK and phospho-Thr172-AMPK (Cell Signaling Technologies). Image densitometry of specific protein bands was performed with ImageJ, developed by Rasband, W.S. at the NIH (http://imagej.nih.gov/ij/), as per the ImageJ instructions.

### AMPK activity assay

AMPK was assayed largely as previously described [27]. Briefly, cells were washed 3X with warm Krebs-Hepes buffer (20 mM Na Hepes, pH 7.4, 118 mM NaCl, 3.5 mM KCl, 1.3 mM CaCl_2_, 1.2 MgSO_4_, 10 mM glucose, 1.2 mM KH_2_PO_4_, 0.1% BSA) and incubated in Krebs-Hepes buffer containing either DMSO, the AMPK inhibitor, Compound C (5 µM), for 1 h at 37°C, or the CaMKK inhibitor, STO-609 (10 µg/ml). Buffer was then aspirated and dishes were placed on ice with immediate addition of 0.25 ml ice-cold lysis buffer (50 mM Tris/HCl, pH 7.4, 50 mM NaF, 5 mM Na pyrophosphate, 1 mM EDTA, 1 mM EGTA, 250 mM mannitol, 1% Triton X-100, 1 mM DTT, protease inhibitors). Cells were scraped and the resulting lysates transferred to microfuge tubes and incubated on ice for 10 minutes. Lysates were then centrifuged for 5 min at 14000 xg and 4°C in preparation for use.

The AMPK assay was composed of a total reaction volume of 25 µl that was incubated for 10 min at 30°C. Each reaction consisted of 2.5 µl lysate assay buffer (62.5 mM Na Hepes, pH 7.0, 62.5 mM NaCl, 62.5 mM NaF, 6.25 mM Na pyrophosphate, 1.25 mM EDTA, 1.25 mM EGTA, 1 mM DTT, and protease inhibitor cocktail (Roche)), 2.5 µl of 100 µM [γ-^32^P]-ATP (1 µCi/µl) in 25 mM MgCl_2_, 2.5 µl of 2 mM AMP in lysate assay buffer, 5 µl of 1 mM SAMS peptide in lysate assay buffer, with either Compound C (5 µM final) or the equivalent volume of DMSO and 12.5 µl cell lysate. The reaction mixture was spotted on P81 phosphocellulose paper which was washed with 1% phosphoric acid, water, and acetone. The radioactivity of the phosphorylated SAMS peptide was quantified by scintillation counting. Non-AMPK-mediated phosphorylation of the SAMS peptide was estimated by performing the AMPK activity assay in the presence of saturating amounts of the AMPK inhibitor, Compound C.

### siRNA experiments

MRC-5 fibroblasts were transfected with 150 pmol of either esiRNA (pooled endoribonuclease-prepared siRNA) specific to AMPK1 (Sigma-Aldrich) or a non-targeting siRNA (Dharmacon) using Oligofectamine per manufacturer's directions. Forty-eight hours after transfection, siRNA-transfected cells were serum-starved for 24 h and then either mock-infected or infected with HCMV (MOI = 3). Samples were harvested at 24 h and 72 h post-infection to monitor AMPK protein knockdown by Western blot. Additional samples were harvested 96 h post-infection to monitor viral titers by standard plaque assay.

### Real-time PCR

Viral and cellular DNA was harvested at various time points post-infection in lysis buffer (100 mM NaCl, 100 mM Tris-HCl, 25 mM EDTA, 0.5% SDS, 0.1 mg/ml proteinase K and 40 µg/ml RNase A), and viral DNA was quantified using the UL26 primer set (below). Quantitative PCR (qPCR) was performed using Fast SYBR green master mix, a model 7500 Fast real-time PCR system and Fast 7500 software (Applied Biosystems). For quantifying viral DNA aU_L_26-HCMV specific primer set was employed: 5_-AACATCGCGTCGGTGATTTCTTGC-3_ (forward) and 5_-ACAGCTACTTTGAAGACGTGGAGC-3_ (reverse), GAPDH 5_-CATGTTCGTCATGGGTGTGAACCA-3_ (forward) and 5_-ATGGCATGGACTGTGGTCATGAGT-3_ (reverse).

### Lactate measurement

Lactate was measured in media samples using the BioProfile 100 Plus/400 (Nova Biomedical), which employs an enzyme dependent amperometric electrode. MRC-5 fibroblasts were cultured in serum free DMEM for 24 h before infection and either mock or HCMV-infected. Lactate excretion into the media was measured over an 18 h interval, starting with a media change. After 18 h, 600 µl of media was removed from each sample dish and analyzed according to the manufacturer's instructions (Nova Biomedical).

### Gene identification information

Further information regarding the genes/ proteins studied in this manuscript can be found at the NCBI Gene Database (http://www.ncbi.nlm.nih.gov/gene). Specific database entries for cellular genes are as follows: AMPK =  PRKAB1, PRKAA1, PRKAG1; CaMKK  =  CAMKK1, CAMKK2; TSC1  =  TSC1; TSC2 =  TSC2; Glut4 =  SLC2A4; ACC1  =  ACACA. The viral genes mentioned include the following from HCMV (also known as Human Herpesvirus 5): UL38  =  UL38; IE1  =  UL123; UL44 =  UL44; pp28  =  UL99.
